# Ethnic differences in complement system biomarkers and their association with metabolic health in men of Black African and White European ethnicity

**DOI:** 10.1093/cei/uxad011

**Published:** 2023-02-01

**Authors:** L M Goff, K Davies, W M Zelek, E Kodosaki, O Hakim, S Lockhart, S O’Rahilly, B P Morgan

**Affiliations:** Department of Nutritional Sciences, School of Population & Life Course Sciences, Faculty of Life Sciences & Medicine, King’s College London, London, UK; Dementia Research Institute Cardiff, School of Medicine, Cardiff University, Cardiff, UK; Dementia Research Institute Cardiff, School of Medicine, Cardiff University, Cardiff, UK; Dementia Research Institute Cardiff, School of Medicine, Cardiff University, Cardiff, UK; Department of Nutritional Sciences, School of Population & Life Course Sciences, Faculty of Life Sciences & Medicine, King’s College London, London, UK; School of Life & Health Sciences, University of Roehampton, London, UK; MRC Metabolic Diseases Unit & Wellcome-MRC Institute of Metabolic Science, University of Cambridge, Cambridge, UK; MRC Metabolic Diseases Unit & Wellcome-MRC Institute of Metabolic Science, University of Cambridge, Cambridge, UK; Dementia Research Institute Cardiff, School of Medicine, Cardiff University, Cardiff, UK

**Keywords:** ethnicity, metabolic health, complement, biomarkers

## Abstract

Inflammation plays a fundamental role in the development of several metabolic diseases, including obesity and type 2 diabetes (T2D); the complement system has been implicated in their development. People of Black African (BA) ethnicity are disproportionately affected by T2D and other metabolic diseases but the impact of ethnicity on the complement system has not been explored. We investigated ethnic differences in complement biomarkers and activation status between men of BA and White European (WE) ethnicity and explored their association with parameters of metabolic health. We measured a panel of 15 complement components, regulators, and activation products in fasting plasma from 89 BA and 96 WE men. Ethnic differences were statistically validated. Association of complement biomarkers with metabolic health indices (BMI, waist circumference, insulin resistance, and HbA1c) were assessed in the groups. Plasma levels of the key complement components C3 and C4, the regulators clusterin and properdin and the activation marker iC3b were significantly higher in BA compared to WE men after age adjustment, while FD levels were significantly lower. C3 and C4 levels positively correlated with some or all markers of metabolic dysfunction in both ethnic groups while FD was inversely associated with HbA1c in both groups, and clusterin and properdin were inversely associated with some markers of metabolic dysfunction only in the WE group. Our findings of increased levels of complement components and activation products in BA compared to WE men suggest differences in complement regulation that may impact susceptibility to poor metabolic health.

## Teaser

Poor metabolic health and obesity in Black African populations contribute to increased susceptibility to disease, including COVID-19. Complement dysregulation has been linked to metabolic health and implicated as a driver of pathology; however, ethnic differences in complement have not been explored. We measured a panel of complement biomarkers in Black African and White European males and found significant differences in complement proteins and activation markers, the latter suggesting a propensity for complement dysregulation.

## Graphical Abstract

**Graphical Abstract:**
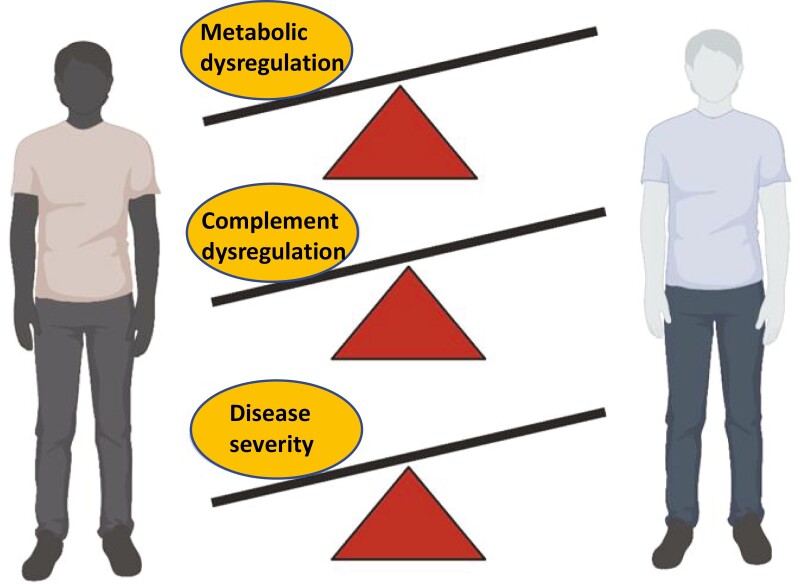

## Introduction

The complement system comprises more than 50 protein receptors and regulators in four major pathways: the classical, lectin, and alternative activation pathways, and the common terminal pathway [1]. It is an integral component of innate immunity with involvement in the elimination of microbes and cellular debris and crosstalk with other immunological, inflammatory, and coagulation processes. Originally characterized as a first-line defence against microbial invaders and eliminator of immune complexes, more recent evidence has implicated the complement system in a diversity of processes, including tissue regeneration, synapse maturation, and lipid metabolism [2–4]. Although a series of positive and negative regulator proteins tightly control the complement system, excessive activation or insufficient control of the system is recognized as a driver of a wide range of inflammatory and immune-regulated disorders including age-related ocular pathologies, cancer, neurodegeneration, and metabolic diseases [5, 6]. Complement dysregulation is also implicated in the maladaptive inflammatory response seen in severe COVID-19; complement is directly activated by the SARS-CoV-2 virus through the lectin and alternative pathways, and anti-viral antibodies can trigger classical pathway activation [7–9]. Complement activation in COVID-19 has been implicated in acute lung injury, pulmonary microvascular thrombosis, and as a trigger of the cytokine storm response [10].

Inflammation is recognized to play a fundamental role in the initiation, development, and progression of several metabolic disorders such as obesity, type 2 diabetes (T2D), and atherosclerosis [11, 12]. A role for the complement system as a driver of inflammation in these contexts has been suggested, likely through adipose tissue-related inflammatory pathways [6, 13]. Complement has important roles in the biology of adipose tissue. Adipocytes are the primary source of factor D, an essential enzyme in the alternative complement pathway; indeed, factor D was independently discovered as a regulator of lipid metabolism, adipsin [14]. Other components and regulators of the alternative pathway, C3, Factor B, properdin, Factor H, and Factor I, are expressed in adipose tissue and plasma levels of these are elevated in people with obesity and/or insulin resistance [15, 16]. C3, the most abundant complement protein, is primarily produced by hepatocytes and is an acute phase reactant; hepatic production is markedly increased in response to pro-inflammatory cytokines. C3 may also provide an important link between inflammation and thrombosis by interacting with fibrin to prolong fibrinolysis in people with diabetes [17].

People of Black African (BA) ethnicity are disproportionately affected by metabolic disorders such as T2D [18, 19]. Obesity or excess adiposity is recognized to be one of the strongest contributors to the development of T2D [20], with risk developing at significantly lower levels of adiposity in people of BA compared to White European (WE) ethnicity [21]. Adipose tissue dysfunction, particularly pro-inflammatory activity, has been implicated as a driver of the insulin resistance that underlies T2D [22]. However, the influence of ethnicity on the complement system has received little attention. To test whether ethnicity impacted complement in a manner that might influence susceptibility to metabolic disorders such as T2D, we measured a panel of complement biomarkers, including activation products, alongside indicators of obesity and metabolic health in men of BA and WE ethnicity resident in the UK.

## Materials and methods

### Study overview and design

An observational study was undertaken principally aiming to compare cardiometabolic risk between men of BA and WE ethnicity. Data and sample collection were conducted at King’s College Hospital, London, UK, between April 2013 and December 2019, as part of the South London Diabetes & Ethnicity Phenotyping Study (Soul-Deep), which was reviewed and approved by the London Bridge National Research Ethics Committee (references: 15/LO/1121 and 12/LO/1859). All participants provided written, informed consent prior to participation.

### Participants

Data included in this study were collected from individuals that were screened for participation in the Soul-Deep study, which is an observational study investigating ethnic differences in the pathophysiology of T2D between men of BA and WE ethnicity [23]. Participants were recruited by advertisements in newspapers, general practices, Facebook, and leaflet distribution. Men aged 18–65 years, either healthy or with prediabetes/T2D were recruited and considered eligible to participate if they: (1) had a body mass index (BMI) of 20–40 kg/m^2^; (2) were of BA or WE ethnicity. Men with a BMI below 20 or above 40 kg/m^2^ were excluded to avoid the inclusion of distinct phenotypes associated with extremes of adiposity in the cohort. Eligibility for participation was determined during an initial assessment which included a screening health questionnaire to report age, date of diabetes diagnosis if applicable, medical history, current medication, and self-declared ethnicity of self, parents, and grandparents; participants were eligible if they self-declared their ethnicity as ‘Black’, ‘Black African’, ‘Black-British’ and were of Black West African heritage, defined as having two parents and four grandparents born in a West African country, according to the United Nations Statistics Division (hereafter, Black African or ‘BA’). Similarly, White European participants were eligible if they self-declared their ethnicity as ‘White’, ‘White-British’, ‘White-European’, ‘White Other’ and were of White European heritage, defined as having two parents and four grandparents born in a Northern, Eastern, or Central European country, according to the United Nations Statistics Division (hereafter, White European or ‘WE’). A fasting blood sample was taken for measurement of full blood count, renal and liver function, HbA1c, lipid profile, sickle cell trait, auto-antibodies (anti-insulin, anti-GAD, and anti-IA2), and complement proteins, which were measured from plasma collected in EDTA tubes. In a sub-sample of the cohort, fasting insulin was measured, and insulin resistance was estimated using the Homeostatic Model Assessment-2 (HOMA2-IR) method [24]. Anthropometric measurements were taken and included height, weight, waist circumference (measured at the midpoint between the lowest rib and iliac crest), and seated blood pressure. Participants were excluded if they showed evidence of liver or kidney damage, determined from a serum alanine aminotransferase (ALT) level of 2.5-fold above the upper limit of the reference range or serum creatinine level above 150 mmol/l, respectively, or tested positive for anti-insulin, anti-GAD or anti-IA2 auto-antibodies, or had documented sickle cell disease.

### Biochemical tests

Plasma glucose concentrations were determined using an automated glucose analyser (Yellow Spring Instruments, 2300 STAT Glucose Analyzer, OH, USA). Serum insulin concentrations were determined by immunoassay using chemiluminescence technology (ADVIA Centaur System, Siemens Healthcare Ltd., Camberley, UK); inter-assay and intra-assay CVs were ≤5.9% and 4.6%, respectively.

Measurement of blood lipids (total-, LDL- and HDL-cholesterol and triglycerides) were conducted in the central clinical pathology laboratory at King’s College Hospital using colorimetric assays on an automated clinical chemistry analyser. HbA1c was measured by boronated affinity binding and high-performance lipid chromatography (Premier Hb9210 analyser, Trinity Biotech, Jamestown, NY, USA); the inter-assay and intra-assay CVs were between 1.28–1.62% and 0.72–1.26%, respectively. Serum insulin concentration was measured by immunoassay using chemiluminescent technology (ADVIA Centaur System, Siemens Healthcare, Camberley, UK); the intra-assay CVs were between 3.2–4.6% and the inter-assay CVs were between 2.6–5.9%.

Plasma complement C3 and C4 were determined in the Clinical Chemistry lab at Addenbrooke’s Hospital Cambridge using laser nephelometry (Beckman-Coulter). The concentrations of complement components (C1q, Factor B (FB), Factor D (FD), C5), regulators (C1 inhibitor (C1inh), Factor H (FH), FH-related proteins 1,2 and 5 (FHR125), FH-related protein 4 (FHR4), Factor I (FI), Properdin, Clusterin), and activation products (iC3b, terminal complement complex (TCC)) were measured using established in-house enzyme-linked immunosorbent assays (ELISA) (Table 1). Maxisorp (Nunc, Loughborough, UK) plates were coated with capture antibody diluted in carbonate buffer (1–5 μg/ml) overnight at 4°C, blocked (1 h at 37°C) with either 2% bovine serum albumin (BSA) or 2% non-fat dried milk (NFM) in phosphate-buffered saline containing 0.5% tween-20 (Sigma Aldrich) (PBS-T), then washed once with PBS-T. Purified protein standards and plasma samples were optimally diluted in 0.2% BSA or 1% NFM in PBS-T according to the individual assay standard operating procedure (SOP) (Table 1) and then added to plates in duplicate (50 µl). Plates were incubated (1.5 h at 37°C), washed with PBS-T, then detection antibody (either unlabelled or labelled with horseradish peroxidase (HRP)) was added and incubated for a further 1 h at 37°C. Plates were washed, incubated with HRP-labelled anti-IgG where required, washed again, and developed using o-phenylenediamine dihydrochloride (OPD, SIGMAFAST™, Sigma–Aldrich). Reactions were stopped with 5% sulphuric acid and absorbances were read at 492 nm. Standards were included on each plate and samples were blinded to eliminate operator bias. Standard curves were fitted using a nonlinear regression model and concentrations in samples were automatically calculated in reference to the curve using GraphPad Prism. Assays were evaluated for sensitivity, reproducibility, and intra- and inter-assay coefficients of variation (CV) in 10 healthy control plasma samples. The intra-assay CV was less than 10% and the inter-assay CV (*n* = 3) was between 1% and 14% in the different assays. Linearity experiments were performed for each assay to determine a suitable dilution factor for plasma samples, chosen as the dilution falling within the linear portion of the log standard curve for all controls.

**Table 1.** Conditions used in ELISA assays.

Assay	Capture antibody	Detection antibody	Standard	Working range (ng/ml)	Sample dilution
*Components*					
C1q	Monoclonal anti-C1q (9H10)	Rabbit anti-C1q	C1q	16–1000	1 in 16 000
FB	Monoclonal anti-FB (JC1)	Monoclonal anti-FB (MBI5)	FB	16–1000	1 in 2000
FD	Rabbit anti-FD	monoclonal anti-FD (D10/4; Hycult)-HRP	FD	1–1000	1 in 10
C5	Monoclonal anti-C5 (4G2)	Rabbit anti-C5	C5	4–500	1 in 2000
*Regulators*					
FH	Monoclonal anti-FH (OX24)	Monoclonal anti-FH (35H9)	FH	16–1000	1 in 4000
FHR125	Monoclonal anti-FHR125 (MBI125)	Monoclonal anti-FH (35H9)	FHR125	4–250	1 in 4000
FHR4	Monoclonal anti-FHR4 (4E9)	Monoclonal anti-FHR4 (clone 150)-HRP	FHR4	16–1000	1 in 20
FI	Monoclonal anti-FI (7B5)	Rabbit anti-FI	FI	32–2000	1 in 200
C1inh	Monoclonal anti-C1inh	Rabbit anti-C1inh	C1 inh	2–100	1 in 6000
Properdin	Monoclonal anti-properdin (clone 1.1.1)	Monoclonal anti-properdin (clone 12.14.2)-HRP	Properdin (CompTech)	78–5000	1 in 200
Clusterin	Monoclonal anti-clusterin (2D5)	Monoclonal anti-clusterin (4E7)	Clusterin	32–2000	1 in 500
*Activation products*					
iC3b	Monoclonal anti-iC3b (Clone 9; Hycult)	Monoclonal anti-iC3b (bH6; Hycult)-HRP	iC3b (CompTech)	16–1000	1 in 300
TCC	Monoclonal anti-TCC (aE11; Hycult)	Monoclonal anti-C8 (E2)-biotin	TCC	10–5000	1 in 40

All antibodies and standard proteins were prepared in house unless otherwise stated. Commercial sources were Hycult (Hycult Biotech; https://www.hycultbiotech.com/) or CompTech (Complement Technology, Inc.; https://www.complementtech.com/). Anti-properdin mAb clone 1.1.1. and clone 12.14.2 were gifted by Prof Santiago Rodriguez de Cordoba, CISC, Madrid. Antibodies shown to be conjugated to HRP or biotin were labelled in-house using commercial kits (Thermo Fisher, UK).

### Statistics

Distribution of datasets was tested for normality using Shapiro–Wilks test. Skewed data were log-transformed prior to applying parametric tests. The significance of differences in the mean values of datasets between the two ethnic groups was tested using an independent samples *t*-test or analysis of covariance (ANCOVA) when controlling for confounders (e.g. age) was required. Relationships between variables of interest were assessed using Pearson’s correlations test. Because these analyses required conducting multiple comparisons, Bonferroni-adjusted significance levels of 0.003 (for comparison of means) and 0.01 (for correlation) were applied to account for the increased possibility of a type-I error. Data are presented as mean with SD, or geometric mean (95% CIs) for log-transformed variables. Statistical analyses were conducted using SPSS 26.0; *P*-values < 0.05 were considered statistically significant.

## Results

### Participant characteristics

The study included 88 BA and 97 WE men. The clinical characteristics of the two ethnic groups are presented in Table 2; allowing for multiple comparisons, the BA men had significantly lower waist circumference than the WE men but there were no ethnic differences in weight, BMI, or insulin resistance (HOMA2-IR). The BA men exhibited significantly lower levels of total cholesterol and triglycerides than the WE men.

**Table 2.** Clinical characteristics of Black African and White European men

	Black African (*n* = 88)	White European (*n* = 97)	*P*
Age, years	41.3 (38.3, 44.5)	48.1 (44.7, 51.7)	**0.018**
Weight, kg	88.2 (85.0, 91.5)	91.9 (88.2, 95.7)	0.316
BMI, kg/m^2^	29.1 ± 4.0	29.3 ± 5.2	0.793
Waist circumference, cm	97.4 ± 12.4	103.6 ± 15.0	**0.003**
Systolic blood pressure, mmHg	130 ± 13	126 ± 12	**0.042**
Diastolic blood pressure, mmHg	79 ± 11	76 ± 10	**0.037**
Total cholesterol, mmol/l	4.25 ± 0.88	4.76 ± 0.85	**<0.001**
LDL-cholesterol, mmol/l	2.57 ± 0.71	2.85 ± 0.75	**0.011**
HDL-cholesterol, mmol/l	1.20 (1.13, 1.27)	1.20 (1.14, 1.26)	0.572
Triglycerides, mmol/l	0.99 ± 0.55	1.30 (1.17, 1.44)	**<0.001**
Fasting glucose, mmol/l	5.6 (5.4, 5.8)	5.8 (5.6, 6.1)	0.403
HOMA2-IR	1.31 (1.14, 1.51)^a^	1.50 (1.25, 1.80)^b^	0.238
HbA1c, mmol/mol	41.7 (40.2, 43.3)	40.2 (38.7, 41.8)	**0.017**

Data presented as mean ± SD, or geometric mean (95% CIs) for variables that required log-transformation prior to testing.

^a^Data available for *n* = 52.

^b^ *n* = 55.

Differences between the two ethnic groups were determined using independent samples *t*-tests. Non-parametric variables were log-transformed before normal tests were carried out on them. Significant differences are highlighted in bold.

### Ethnic differences in complement components

**Figure 1.**
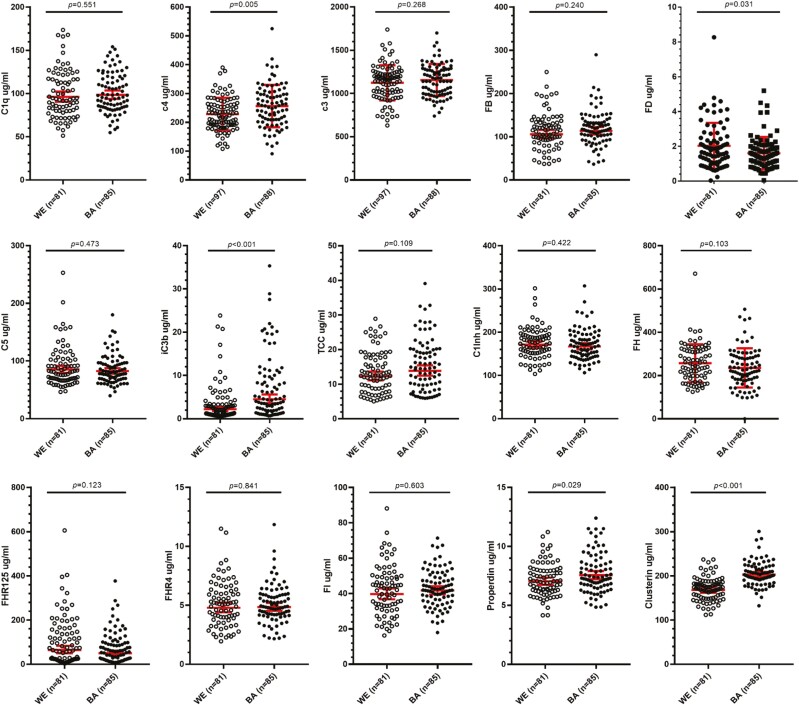
Comparison of complement biomarker levels between White European (WE) and Black African (BA) males. Fifteen complement biomarkers were measured. C3 and C4 were measured by nephelometry in 97 WE and 88 BA samples. The other 13 biomarkers were measured by ELISA in 81 WE and 85 BA samples. Significance of differences between the groups of the unadjusted means are indicated

The unadjusted means for the complement markers in the BA (C3, C4 *n* = 88; others *n* = 85) and WE (C3, C4 *n* = 97; others *n* = 81) men are shown in Table 3 and Fig. 1. Significance of differences between groups for each biomarker is shown uncorrected and corrected for age differences between the ethnic groups; the Bonferroni-adjusted *P*-value of 0.003 was used to indicate significance. After adjustment for age, the abundant complement component C4, the regulator clusterin, and the activation marker iC3b were significantly higher in BA versus WE men. TCC, the other activation marker measured, was also increased in BA men, although the difference was not significant.

**Table 3.** Complement marker levels of Black African and White European men

	Black African		White European		*P*	P
	*n*		*N*		Unadjusted	Adjusted for age
*Components*						
C1q µg/ml	85	98.5 (93.7, 103.6)	81	96.2 (90.6, 102.2)	0.551	0.791
C4 µg/ml	88	257.0 ± 74.0	97	229.0 ± 57.0	0.005	<0.001
C3 µg/ml	88	1160 ± 190.0	97	1120 ± 200.0	0.268	0.030
FB µg/ml	85	113.8 (105.4, 122.9)	81	105.8 (96.7, 115.8)	0.240	0.215
FD µg/ml	85	1.60 ± 0.94	81	1.95 ± 1.12	0.031	0.017
C5 µg/ml	85	82.3 (77.6, 87.4)	81	85.3 (79.3, 91.8)	0.473	0.838
*Activation products*						
iC3b µg/ml	85	4.51 (3.60, 5.66)	81	2.19 (1.77, 2.71)	<0.001	<0.001
TCC µg/ml	85	13.97 (12.55, 15.54)	81	12.31 (11.13, 13.63)	0.109	0.238
*Regulators*						
C1inh µg/ml	85	167.1 (159.9, 174.5)	81	171.3 (163.8, 179.2)	0.422	0.399
FH µg/ml	85	222.4 (205.1, 241.3)	81	243.4 (226.3, 261.9)	0.103	0.185
FHR125 µg/ml	85	49.4 (39.9, 61.1)	81	64.8 (50.1, 83.8)	0.123	0.190
FHR4 µg/ml	85	4.85 (4.52, 5.20)	81	4.80 (4.41, 5.22)	0.841	0.840
FI µg/ml	85	43.1 ± 10.7	81	42.1 ± 14.0	0.603	0.124
Properdin µg/ml	85	7.58 (7.23, 7.94)	81	7.04 (6.74, 7.36)	0.029	0.031
Clusterin µg/ml	85	203.1 (197.3, 209.0)	81	167.9 (162.0, 174.0)	<0.001	<0.001

Data presented as mean ± SD, or geometric mean (95% CIs) for transformed variables. Differences between the two ethnic groups were determined using independent samples *t*-tests or analysis of covariance (ANCOVA) when controlling for confounders. Non-parametric variables were log-transformed before normal tests were carried out on them. P^1^ unadjusted comparison of means; P^2^ age-adjusted comparison of means. Significant differences are highlighted in bold.

### Associations between complement components and metabolic health

Associations between complement biomarkers and markers of metabolic health are shown in Table 4; the Bonferroni-adjusted *P*-value of 0.010 was used to indicate significance. In both the WE and BA groups, complement C3 showed significant positive associations with BMI, HOMA2-IR, and HbA1c. C4 was significantly positively associated with waist circumference, BMI, and HOMA2-IR only in the WE men. In the BA men, C4 was positively associated with HbA1c. Clusterin was significantly negatively associated with waist circumference in WE but not BA men, and properdin was negatively associated with HbA1c in the WE group only. The activation marker iC3b was not significantly associated with any markers of metabolic health in either ethnic group.

**Table 4.** Associations between complement markers and metabolic health

	Black African		White European	
	*r*	*p*	r	*P*
*C3 (BA n = 88; WE n = 97)*				
Age*	0.403	<0.001	0.361	<0.001
BMI	0.571	<0.001	0.625	<0.001
Waist	0.585	<0.001	0.660	<0.001
HOMA2-IR*^a^	0.561	<0.001	0.630	<0.001
HbA1c*	0.516	<0.001	0.350	<0.001
*C4 (BA n = 88; WE n = 97)*				
Age*	0.362	0.001	0.332	0.001
BMI	0.195	0.068	0.303	<0.001
Waist	0.234	0.029	0.382	<0.001
HOMA2-IR*^a^	0.200	0.156	0.339	0.011
HbA1c*	0.462	<0.001	0.240	0.018
iC3b* (BA n = 85; WE n = 81)				
Age*	–0.145	0.184	0.107	0.343
BMI	–0.028	0.800	0.138	0.219
Waist	–0.068	0.537	0.197	0.081
HOMA2-IR*^a^	0.149	0.297	0.130	0.352
HbA1c*	–0.108	0.326	–0.173	0.124
Clusterin* (BA n = 85; WE n = 81)				
Age*	–0.008	0.943	–0.003	0.976
BMI	–0.046	0.673	–0.264	0.017
Waist	–0.062	0.572	–0.290	0.009
HOMA2-IR*^a^	0.112	0.433	–0.240	0.083
HbA1c*	0.072	0.510	–0.190	0.090
Properdin* (BA n = 85; WE n = 81)				
Age*	0.187	0.087	–0.110	0.328
BMI	0.231	0.033	0.056	0.620
Waist	0.124	0.261	–0.028	0.803
HOMA2-IR*^a^	0.059	0.682	–0.149	0.288
HbA1c*	-0.039	0.722	–0.389	<0.001
*FD (BA n = 85; WE n = 81)*				
Age*	–0.169	0.123	–0.034	0.762
BMI	0.011	0.917	0.145	0.199
Waist	0.013	0.909	0.126	0.270
HOMA2-IR*^a^	0.128	0.370	0.113	0.419
HbA1c*	–0.240	0.027	–0.227	0.043

Correlation coefficients determined using Pearson’s correlation.

*Data were log-transformed before analysis to achieve a normal distribution.

^a^Data available for BA *n* = 52 and WE *n* = 55.

## Discussion

Increased complement activation is well described in metabolic disorders such as obesity and T2D, which are disproportionately prevalent in people of BA ethnicity. However, to date, ethnic differences in complement system proteins and propensity to activate have not been explored. We tested whether there were ethnic differences in the complement system between men of BA and WE ethnicity that might impact the risk of disordered metabolic health. We analysed plasma samples from highly phenotyped BA and WE men collected as part of the Soul-Deep study of ethnic differences in the pathophysiology of T2D [23]. Fifteen complement biomarkers were measured, compared between the ethnic groups and correlated with metabolic health parameters. We found significantly higher expression of the complement components C3 and C4, regulators clusterin, and properdin and the activation marker iC3b, and lower expression of FD, in BA compared to WE men. C3 and C4 are the most abundant complement proteins and the only ones routinely measured, usually for monitoring disease activity in lupus or other autoimmune diseases. Increased C3 and C4 may confer a higher capacity for complement activation in the BA group, although this is unlikely to be responsible for complement dysregulation evidenced by increased levels of the activation marker iC3b in this group. Properdin is a positive regulator of the complement amplification loop; higher properdin levels in BA men further support a predilection for activation. FD is an essential enzyme of the amplification loop with known roles in lipid metabolism; uniquely among complement proteins FD is primarily synthesized in adipose tissue [14–16]. Obesity and the associated increased adipose tissue mass is associated with elevated FD [25, 26]; lower levels of FD in BA men may thus be related to lower adipose tissue mass in this group. Clusterin is a multifunctional protein with guises that include chaperone, lipoprotein (ApoJ), and negative regulator of the terminal complement pathway [27]; higher levels of clusterin in BA males predict tighter control of the terminal pathway, a prediction supported by finding no difference in TCC between groups.

An impact of obesity or other metabolic conditions on complement levels has been reported. In the large CODAM study, adiposity and BMI were positively associated with plasma levels of C3, FD, properdin, and the activation marker C3a [28]. Plasma clusterin was increased in overweight and obese individuals, correlated with BMI, and was suggested as a surrogate obesity marker [29]; properdin levels and indicators of complement activation were increased in donors with a family history of T2D and suggested as markers of future risk [30, 31]. Obesity and other metabolic health issues and BA ethnicity are thus impacting complement homeostasis in similar ways. In our study, BMI was not different between the ethnic groups, though the WE group was older, and had greater waist circumference and higher cholesterol and triglycerides, while the BA group had higher plasma HbA1c. These small and inconsistent differences between the groups are unlikely to be responsible for the observed differences in complement biomarkers between the ethnic groups. Indeed, BA men in the UK have similar prevalence rates of obesity to WE men yet are disproportionately affected by T2D and other metabolic disorders [18].

There has been a wealth of research in recent years investigating the potential role of complement dysregulation in COVID-19 [7-10]. However, none have compared levels of complement biomarkers in COVID-19 patients from different ethnic groups, despite unequivocal evidence that people from minority ethnic backgrounds experience greater COVID-19 severity and poorer outcomes. The demonstration that, compared to WA men, BA men have elevated levels of multiple complement biomarkers compatible with a more activating phenotype, provokes us to suggest that increased propensity for dysregulated complement activation may contribute to increased COVID-19 disease severity in BA men. Interestingly, genetic studies in populations of BA descent have shown a high prevalence of gene variants in critical complement regulators that are associated with increased complement activation and a higher risk of thrombotic microangiopathy and multi-organ injury, both of which are common complications in severe COVID-19 [32]. If dysregulated complement activation were born out as a driver of COVID-19 severity in BA men, anti-complement therapies may prove to be particularly effective in reducing the severity of COVID-19 in BA patients, particularly in those with underlying health conditions [28].

Our study has several strengths and limitations to acknowledge. To our knowledge, this is the first investigation of ethnic differences in complement system components and activation products between a BA and WE population. The ethnic groups were well matched for BMI which minimizes the risk of confounding. However, the study included only men, which may limit the generalizability of our findings, and our cohort was restricted to those with a BMI between 20 and 40 kg/m^2^, preventing us from exploring the implications for ethnic differences in complement of more extreme levels of adiposity. Another limitation is our lack of genetic data in this cohort, which limits our ability to understand the role of genetics in driving the complement differences we have observed.

In conclusion, men of BA ethnicity exhibit increased expression of complement components and activation products compared to WE men, beyond that explained by adiposity. A propensity for complement over-activation may contribute to the greater risk of metabolic disorders in populations of BA ethnicity.

## Data Availability

Data are available upon request from the corresponding author.

## Abbreviations

BA: black African
BMI: body mass index
CV: coefficient of variation
HbA1c: glycated haemoglobin
HDL: high density lipoprotein
T2D: type 2 diabetes
WE: white European

## Permission to reproduce material from other sources

Not applicable to this submission.

## Clinical trial registration

Not applicable to this submission.
